# Palliative care in Africa: a global challenge

**DOI:** 10.3332/ecancer.2014.493

**Published:** 2014-12-11

**Authors:** Christian R Ntizimira, Jean Luc Nkurikiyimfura, Olive Mukeshimana, Scholastique Ngizwenayo, Diane Mukasahaha, Clare Clancy

**Affiliations:** 1 OhioHealth HomeReach Hospice, Kobacker House, 800 McConnell Drive, Columbus, Ohio 43214-3463, USA; 2 Teaching Hospital of Kigali (CHUK), 1024 Rue de la Paix, Kigali City, Kigali, PO Box 655, Rwanda; 3 Kibagabaga Hospital, PO Box 6260, Kigali City, Rwanda; 4 Rwanda Biomedical Centre, PO Box 640, Kigali, Rwanda; 5 74–2400 Tell Place, Regina, SK S4V 3E3, Canada

**Keywords:** palliative care, global challenge, pain, education, implementation, Africa, Rwanda

## Abstract

We are often asked what challenges Rwanda has faced in the development of palliative care and its integration into the healthcare system. In the past, patients have been barred from accessing strong analgesics to treat moderate to severe pain, but thanks to health initiatives, this is slowly changing. Rwanda is an example of a country where only a few years ago, access to morphine was almost impossible. Albert Einsten said ‘in the middle of difficulty lies opportunity’ and this sentiment could not be more relevant to the development of palliative care programmes. Through advocacy, policy, and staunch commitment to compassion, Rwandan healthcare workers are proving how palliative care can be successfully integrated into a healthcare system. As a global healthcare community, we should be asking what opportunities exist to do this across the African continent. Champions of palliative care have a chance to forge lasting collaborations between international experts and African healthcare workers. This global network could not only advocate for palliative care programmes but it would also help to create a culture where palliative care is viewed as a necessary part of all healthcare systems.

Palliative care has been defined by the World Health Organisation (WHO) as ‘an approach that improves the quality of life of patients and their families facing the problems associated with life-threatening illness, through the prevention and relief of suffering by means of early identification and impeccable assessment and treatment of pain and other problems, physical, psychosocial, and spiritual’ [1]. Palliative care is a relatively new discipline in Africa and its development is hampered by the fact that the concept of pain management is not integrated into healthcare systems. Only four of 53 African countries have integrated palliative care into healthcare policy or used it as part of a strategic plan focusing on cancer treatment. These include Kenya, South Africa, Tanzania, and Uganda, while Rwanda and Swaziland have taken a different approach by developing stand-alone national palliative care policies [2].

Now that there is increasing awareness of palliative care, healthcare professionals across the continent have an opportunity to examine access to treatment. We can strengthen our efforts to share experiences and create a vast network of countries and organisational partners which use a global language on palliative care. Palliative care presents a global challenge, with all ministries of health burdened by patients in moderate to severe pain. This is especially true in sub-Saharan Africa, where an estimated 24.7 million people were living with Human Immunodeficiency Virus/Acquired Immune Deficiency Syndrome (HIV/AIDS) in 2013, accounting for 70% of the global disease burden, with more than 1.4 million new infections reported in 2013 alone [3]. There were over 700,000 new cancer cases and nearly 600,000 cancer-related deaths in Africa in 2007 [4] and cancer rates on the continent are expected to grow by 400% over the next 50 years [5].

## Challenges versus opportunities

Evidence-based research clearly demonstrates that there is a need and high demand for a holistic approach to palliative care on the continent. It is time to show all Africans why this is a global issue and how palliative care can improve the quality of life for millions of patients with cancer and HIV/AIDS who suffer from moderate to severe pain. The WHO public health model touts integrated palliative care in public health institutions to allow a comprehensive approach to treatment. Through political advocacy and public awareness, the necessity of integrated palliative care can be better understood.

**Policy:** Two out of 53 countries (Rwanda, Swaziland) have a standalone national policy, strategic and implementation plan while four countries (Uganda, Kenya, Tanzania, South-Africa) have integrated palliative care into public health services. There is a need for strong laws to make palliative care an imperative for patients who are facing chronic or end-of-life illness. Just as each healthcare system is fraught with its own challenges, so too is it necessary to use tailored approaches for palliative care.

**Drug availability:** In many African health care systems the concept of Opiophobia, or a fear of Opioids, is prevalent. In some cases, misunderstanding and the fear of addiction have led healthcare workers to provide restricted treatment, leaving in patients in unnecessary pain at the end of life.

Pain and psychosocial distress are highly prevalent among patients with serious chronic diseases such as cancer and HIV/AIDS, greatly reducing their quality of life (Harding 2012; Teunissen 2007; Uwimana 2007). Pain management would help to combat the psychological distress that can go hand-in-hand with a terminal or chronic illness.

Palliative care focuses on the relief of suffering of any kind and on maximizing the quality of life of patients and their families (Harding 2005b; WHO 2013). Thus, palliative care is widely considered a human right (Brennan 2007; Gwyther 2009). Yet, it is rarely accessible in resource-limited settings (Connor 2012; Farmer 2010; Harding 2005a; Krakauer 2010b; Lamas 2012; Uwimana 2007), due to multiple related factors, including lack of opioid availability, provider training, and decentralisation of key services from the central hospital level to the health centre or patient home. Therefore, patients with advanced chronic illnesses typically are discharged from the healthcare system and return home with no follow-up treatment when they are most likely to have severe or worsening symptoms, when they are the most physically and socio-economically vulnerable, and when their families are under the greatest stress (Harding 2009; Vogel 2011).

**Education:** A general lack of awareness surrounding palliative care issues comes at a great cost. Freedom from pain is a universal right, crossing cultures, ethnicities, beliefs, and religions, and must be rooted in the concept of humanity. We need to understand how this global issue impacts country populations. African leaders in particular must be educated on palliative care through evidence-based research. According to the United Nations World Population Prospects, the population will increase to 1.4 billion in 2025 and 1.9 billion in 2050.

One in every three children in the world will be born in Sub-Saharan Africa. Africa is also the only continent in the world where population is expected to keep growing beyond 2100. While this presents a challenge, there is an amazing opportunity to educate the public and involve local communities.

Palliative care champions in Africa are working to demonstrate how morphine access can be appropriately integrated into healthcare systems. Various initiatives in Africa have not only improved patients’ quality of life, but these programmes have also helped to take some of the burden off of healthcare systems. Here are some of the most notable people working in the field:

The founder of Hospice Africa and Director of International Programmes at Hospice Africa Uganda (HAU), Dr. Anne Merriman, has been nominated for a Nobel Peace prize. Dr. Merriman was nominated for work that has contributed to palliative care development in Africa. She introduced palliative care in Singapore, helped to establish a formula for liquid oral morphine, established Hospice Africa, lobbied for the legalisation of morphine in Uganda, developed a centre for excellence in palliative care for Africa in Hospice Africa Uganda, developed the Institute for Hospice and Palliative Care in Africa and was a founding member of the Palliative Care Association of Africa, the African Palliative Care Association and the palliative care unit at Makerere University (Hospice Africa Uganda March 2014 Newsletter). Uganda and the rest of Africa will always be grateful for all she has done and achieved in the past years, with health care workers continuing to learn about the positive effects of home-based care from her experiences.

Dr. Faith Mwangi-Powell is the senior programme officer for global advocacy with the International Palliative Care Initiative of the Open Society Foundations. She was the founding executive director of the African Palliative Care Association, a pan-African organisation supporting the scale-up of palliative care across Africa, where she served for eight years, overseeing the development of palliative care in over 20 African countries. She demonstrated a strong leadership in advocacy in palliative care among leaders in Africa, and her skills and knowledge have helped to improve the quality of life of many patients and their families across different countries.

Dr. Zipporah Ali is the national coordinator of Kenya Hospices and Palliative Care Association (KEHPCA) and a board member of APCA (African Palliative Care Association). She was among those who received the APCA award for her contributions to palliative care in Africa and her work on the inaugural board of APCA. She is also a member of the board of the Kenya Cancer Association. Africa should be proud of this woman for her dedication to ending chronic pain, having helped create a palliative care programme at the Kenya Ministry of Health.

There are many activists and palliative care advocates in Africa who are dedicating their lives to combating unnecessary pain. African healthcare workers have an opportunity to become an example for how successful pain management systems can be integrated into the public healthcare of developing countries. Patients who are facing life-limiting illnesses, healthcare workers who are on the front lines of the fight against cancer and HIV/AIDS as well as policy makers must work together to improve the quality of life for those in pain.

## Conclusion

The implementation of palliative care is a global health challenge, which has implications not only for health-care systems but for various layers of our society. Millions of patients, especially in low- and middle-income countries, experience unnecessary suffering and pain without access to morphine. This has social and psychological effects on families dealing with the end-of-life care of a loved one. The consequence of the ‘boomerang effect’ will impact our economy, our lifestyle, and attitude, which will in turn affect future generations. The enhanced WHO public health model provides an effective strategy for integrating palliative care in all African countries. It is up to this generation of health-care providers to offer patients this multi-sectorial and multidisciplinary approach in Africa [6].

I (CN) still remember a conversation I had with one of my patients. He was 60 years old, well educated, and diagnosed with penile cancer during his last days. When we discussed his advanced directives, he said: “Death is still the elephant in the room in Rwanda.” I replied: “Yes, but how can we help?” After looking at me, he offered a small smile and answered: “Either we eat the elephant which will take time or chase it out, but you can be sure it will destroy the room.” Death is a reality of the medical profession, but pain doesn’t have to be.
